# Supplementary material for: Insecticidal Toxicities of Three Main Constituents Derived from Trachyspermum ammi (L.) Sprague ex Turrill Fruits against the Small Hive Beetles, Aethina tumida Murray
Source: Molecules. 2020 Mar 1;25(5):1100. doi: 10.3390/molecules25051100 (PMC7179092; doi:10.3390/molecules25051100)
Supplement: Supplementary file 1 [file molecules-25-01100-s001.pdf]

Supplementary data (Online)

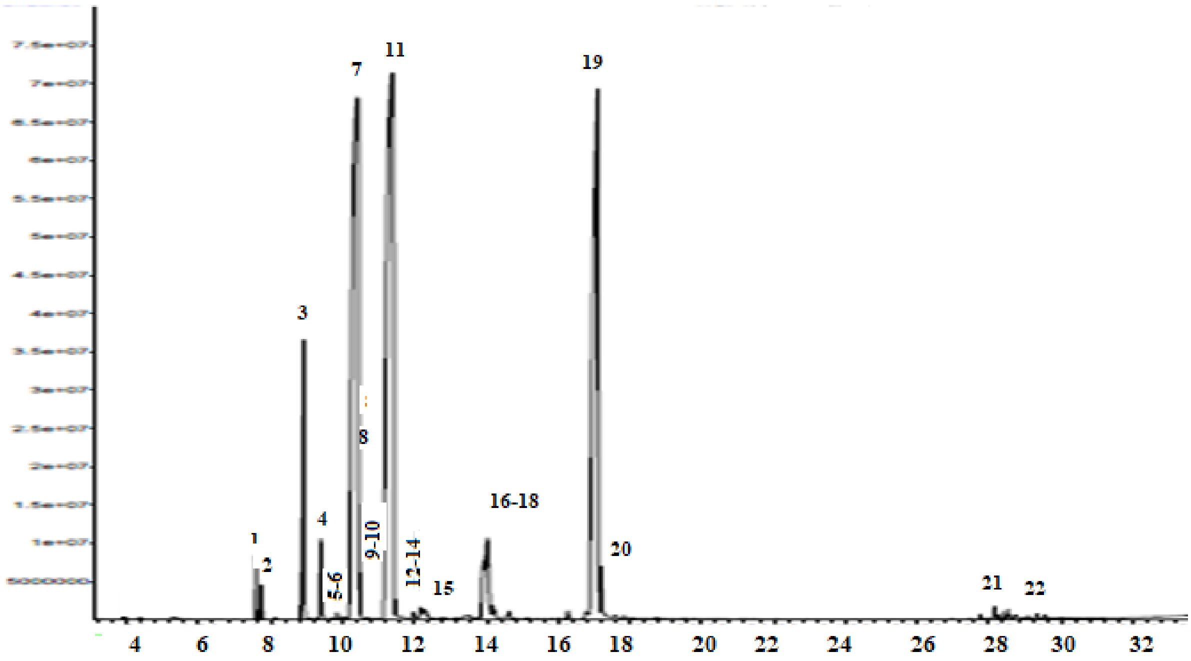

Figure S1. GC-MS chromatogram of *Trachyspermum ammi* fruits essential oil

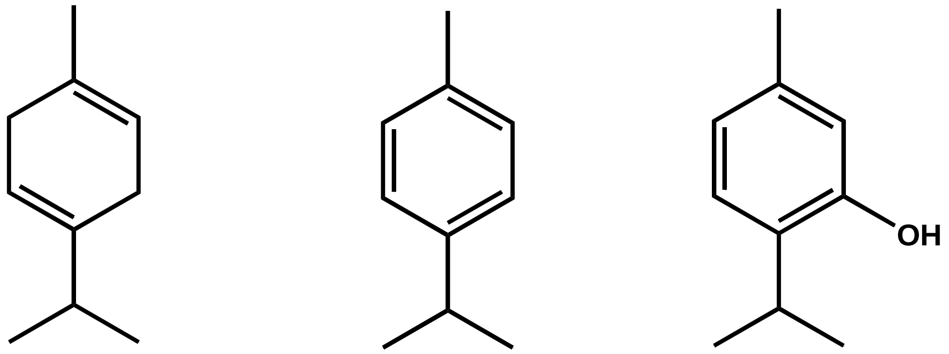

1 **2** **3**

Figure S2. Chemical structures of γ-terpinene (**1**), *p*-cymene (**2**) and thymol (**3**).

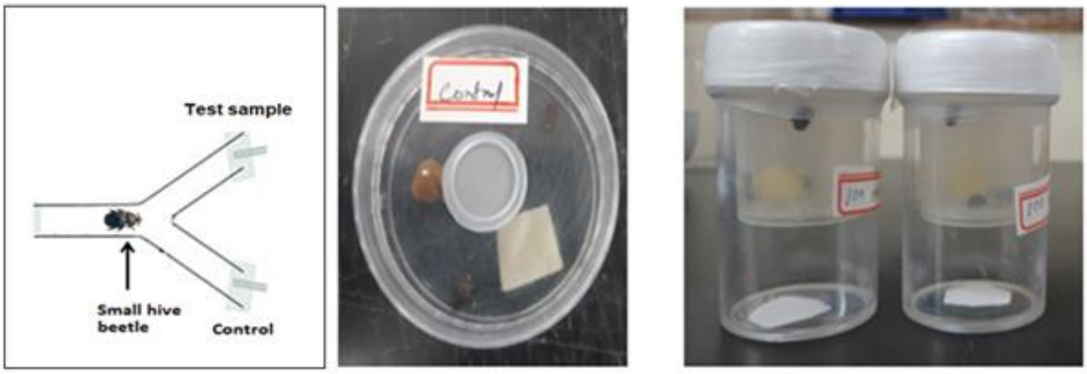

(A) (B) (C)

Figure S3. Bioassay-set ups, (A): Y-tube olfactometer; (B): Contact toxicity; (C): Fumigant toxicity
